# Genome Sequence of *Porphyromonas gingivalis* Strain AJW4

**DOI:** 10.1128/genomeA.01304-15

**Published:** 2015-11-05

**Authors:** Gary Xie, Ryan P. Chastain-Gross, Myriam Bélanger, Dibyendu Kumar, Joan A. Whitlock, Li Liu, William G. Farmerie, Hajnalka E. Daligault, Cliff S. Han, Thomas S. Brettin, Ann Progulske-Fox

**Affiliations:** a Bioenergy and Biome Sciences (B-11), Bioscience Division, Los Alamos National Laboratory, Los Alamos, New Mexico, USA; b Department of Oral Biology, University of Florida, Gainesville, Florida, USA; c Center for Molecular Microbiology, University of Florida, Gainesville, Florida, USA; d Interdisciplinary Center for Biotechnology Research, University of Florida, Gainesville, Florida, USA

## Abstract

*Porphyromonas gingivalis* is associated with oral and systemic diseases. Strain-specific *P. gingivalis* invasion phenotypes have been correlated with disease presentation in infected laboratory animals. Here, we present the genome sequence of AJW4, a minimally invasive strain, with a single contig of 2,372,492 bp and a G+C content of 48.27%.

## GENOME ANNOUNCEMENT

*Porphyromonas gingivalis* is an anaerobic bacterium (1) found in the oral tissues of chronic periodontitis patients (2, 3) and selected nonoral tissues of systemic disease patients (4–6). Invasion of nonphagocytic host cells may be a mechanism by which *P. gingivalis* promotes oral and systemic disease (7). *In vitro* studies have shown variation among strain invasion phenotypes (8–10). Animals inoculated with highly invasive strains displayed oral (11) and vascular (12) disease, while invasion-deficient strains generated no disease. Presently, the genomic sequences and corresponding invasion phenotypes are available for only three highly invasive *P. gingivalis* strains, W83, ATCC 33277, and A7436 (13–15). AJW4 is the only widely available minimally invasive strain of *P. gingivalis* (7, 8). Isolated at the State University of New York-Buffalo in 1988 (16), AJW4 exhibited 1% average *P. gingivalis* invasion efficiency in an *in vitro* analysis (8). Microarray analyses comparing AJW4 and W83, an invasive strain, detected 120 putative genes in W83 that were heavily modified or absent in AJW4 (7), including sequences linked to invasion capacity (17). This study was undertaken to determine the complete genome sequence of AJW4 and expedite the identification of the genetic components required for successful invasion by *P. gingivalis.*

*P. gingivalis* strain AJW4 was obtained from B. G. Loos (State University of New York at Buffalo, NY) and grown as previously described (18). Genomic DNA was obtained using the Wizard genomic DNA (gDNA) purification kit (Promega) and processed to generate shotgun and 3-kb paired-end libraries, which were sequenced using the 454 Life Sciences GS-20 instrument (Roche) (19). A total of 546,865 reads of 143,355,863 bases, with an average read length of 262 bp, were generated.

The GS-20 reads were assembled using Velvet version 0.7.63 (https://www.ebi.ac.uk/~zerbino/velvet/) (20) and Newbler version 2.3 (Roche) (19). Gaps between contigs were closed by editing in Consed (http://www.phrap.org/consed/consed.html) (21–23) and by PCR-augmented Sanger sequencing. The genome was annotated using the RAST (http://metagenomics.anl.gov) (24) and IMG-ER (http://img.jgi.doe.gov/er) (25) servers and then amended using the Gene Prediction Improvement Pipeline software (https://geneprimp.jgi-psf.org) (26).

The genome of *P. gingivalis* AJW4 has approximately 59-fold coverage and contains a single contig of 2,372,492 bp (G+C content, 48.27%). A total of 2,073 genes were annotated, which included 2,006 predicted coding sequences (CDSs), 53 tRNAs, 20 rRNAs, and 1 transfer-messenger RNA (tmRNA). There are 231 subsystems in the genome; also, 193 protein metabolism, 158 cofactors, vitamins, prosthetic groups, and pigments, 64 RNA metabolism, 87 DNA metabolism, 96 carbohydrate, and 19 membrane transport subsystem features were observed.

The annotated *P. gingivalis* AJW4 genome was compared to that of *P. gingivalis* strains W83, ATCC 33277, and TDC60 using RAST (24) and IMG-ER (25). All-to-all BLASTP comparisons of the predicted protein sequences showed that AJW4 possesses 154 strain-specific CDSs, of which 123 are annotated as hypothetical proteins. Notably, AJW4 and W83 contain similar quantities of DNA base pairs and predicted genes, suggesting that the widespread loss of genetic information is not a basis for the minimally invasive AJW4 phenotype.

The availability of the AJW4 genome aids explorations of the genomic elements necessary for the invasion of host cells by *P. gingivalis*, a key aspect of its association with oral and systemic pathologies.

### Nucleotide sequence accession number.

This genome sequencing project was deposited in GenBank under the accession no. CP011996. The version described is the first version.
